# Low intensity stimulation of aortic baroreceptor afferent fibers as a potential therapeutic alternative for hypertension treatment

**DOI:** 10.1038/s41598-022-15761-y

**Published:** 2022-07-18

**Authors:** Ibrahim M. Salman, Omar Z. Ameer, Sheridan McMurray, Sarah F. Hassan, Arun Sridhar, Stephen J. Lewis, Yee-Hsee Hsieh

**Affiliations:** 1 Department of Pharmaceutical Sciences, College of Pharmacy, Alfaisal University, Riyadh, 12714 Saudi Arabia; 2 Division of Pulmonology, Allergy and Immunology, Department of Pediatrics, School of Medicine, Case Western Reserve University, Cleveland, OH USA; 3 Galvani Bioelectronics, Stevenage, Hertfordshire UK; 4 Electrical Stimulation Center, Case Western Reserve University, Cleveland, OH USA; 5 Division of Pulmonary, Critical Care, and Sleep Medicine, School of Medicine, Case Western Reserve University, Cleveland, OH USA

**Keywords:** Neuroscience, Physiology, Pathogenesis, Cardiac device therapy

## Abstract

Carotid baroreceptor stimulation has been clinically explored for antihypertensive benefits, but neuromodulation of aortic baroreceptor afferents remains unexplored for potential translation into the clinic. Published studies have used supramaximal stimulations, which are unphysiological and energy inefficient. The objective of the present study was to identify optimal low-charge nerve stimulation parameters that would provide a clinically-relevant (20–30 mmHg) decrease in mean arterial pressure (MAP) in anesthetized spontaneously hypertensive rats. Stimulations of 20 s were delivered to the left aortic depressor nerve (ADN) of these rats using low ranges of pulse amplitudes (≤ 0.6 mA), widths (≤ 0.5 ms) and frequencies (≤ 5 Hz). We also assessed the effects of continuous (20 s) *versus* intermittent (5 s ON/3 s OFF and 5 s ON/3 s OFF for 20 s) stimulation on MAP, heart rate (HR), mesenteric (MVR) and femoral (FVR) vascular resistance using low (5 Hz) and high (15 Hz) frequencies. Lower pulse amplitudes (0.2 mA) produced 9 ± 2 to 18 ± 2 mmHg decreases in MAP. Higher pulse amplitudes (0.4 mA) produced a median MAP reduction of 28 ± 4 mmHg at 0.2 ms and 5 Hz, with no added benefit seen above 0.4 mA. Continuous and intermittent low frequency stimulation at 0.4 mA and 0.2 ms produced similar sustained decreases in MAP, HR, MVR and FVR. Continuous high frequency stimulation at 0.4 mA and 0.2 ms produced larger reductions in MAP, HR, MVR and FVR compared with all low frequency and/or intermittent high frequency stimulations. We conclude from these findings that “low intensity intermittent” electrical stimulation is an effective alternate way for neuromodulation of the aortic baroreceptor afferents and to evoke a required restoration of MAP levels in spontaneously hypertensive rats. This approach enables low energy consumption and markedly lowers the excessive decreases in MAP and hemodynamic disturbances elicited by continuous high-charge injection protocols.

## Introduction

Chronic hypertension affects more than one billion people worldwide^1^ and is a major risk factor for sudden cardiac death^2^. This common clinical condition increases the risk for heart disease and stroke, both of which are leading causes of morbidity and mortality in this patient population^3^. Currently, dietary modification, salt restriction, regular exercise and antihypertensive medications remain the treatment(s) of choice for hypertensive subjects^4^. However, resistant hypertensive patients, who fail to adequately respond to available treatment options, remain under-treated and therefore highly predisposed to increased risk of cardiovascular events. As a result, there is still a persistent need for novel therapeutic strategies aimed at for instance, lowering sympathetic nerve activity (SNA), to decrease arterial blood pressure (ABP) within the hypertensive population.

In the recent years, device-based neurostimulation has gained popularity as a novel means to control hypertension. This intervention alters central autonomic drive by directly enhancing the activity of baroreceptor afferent neurons, a set of nerve fibers that are frontline sensors that respond to fluctuations in ABP and relay this information directly to the nucleus tractus solitarius (NTS) in the brainstem, which in turn sends information to other cardiovascular control centers within the brainstem and higher regions^5^. The results of activating baroafferents include inhibition of sympathetic drive to the heart and vasculature and increased vagal parasympathetic drive to the heart, which together translate into decreases in peripheral vascular resistances, cardiac output, heart rate and therefore ABP^5–7^. The overall process mimics spontaneous activation of the baroreceptor reflex, albeit bypassing the process of ABP-induced activation of mechanosensitive baroreceptors in the carotid sinus and the aortic arch. This therefore enables a defined control of afferent input into the central nervous system (CNS) and fine-tunes the magnitude of ABP drop^8^. Indeed, baroreflex activation therapy (BAT) targeting the carotid sinus, via the implantable first-generation *Rheo Hypertension Therapy System* and then the second-generation *Barostim Neo*, has produced sustained decreases in SNA and ABP in humans^9,10^, with some preclinical studies^11,12^ and controlled clinical trials^13–15^ having been undertaken to grant the devices approval to treat patients with resistant hypertension. Available data on this innovative treatment approach^14–16^ indicate that there is an urgent need to improve the safety and efficacy profiles. For instance, published experimental and clinical studies employ large current/voltage amplitudes, longer pulse widths or ultrahigh frequencies during electrical stimulation^10,17,18^. These highly energy inefficient neuro-modulation methods, which in some cases lead to excessive decreases in ABP^18–20^, and which compromise the efficacy and the longevity of this stimulation paradigms, warrant further research studies that are designed to optimize the charge parameters.

Although carotid baroreceptor stimulation has been clinically explored for antihypertensive benefits, aortic baroreceptor afferents modulation, on the other hand, remains untouched for clinical translation. Published evidence suggests that aortic baroreceptors^21–23^ and their afferent fibers^24^ display higher pressure sensitivity than carotid baroreceptors, possibly due to the more important functional role they play in control and maintenance of systemic ABP. This potentially makes the aortic baroreceptors a superior novel neural target for ABP modulation and therefore an important alternative avenue for therapeutic intervention in hypertension.

Direct electrical stimulation of the baroreceptor reflex arc in spontaneously hypertensive rats (SHR), via activation of baroreceptor afferents within the aortic depressor nerve (ADN), may bring in novel way of developing and enhancing BAT by providing a safe and effective stimulus paradigm. Our main goal was therefore to investigate the aortic baroreceptor afferent stimulation in SHR as a viable target for ABP modulation and to identify optimal minimal charge stimulation parameters that would provide a clinically relevant drop in mean arterial pressure (MAP) of 20 – 30 mmHg as reported previously^10,16,25,26^.

## Results

### Study 1: Optimization of ADN stimulation parameters

**Figure 1:**
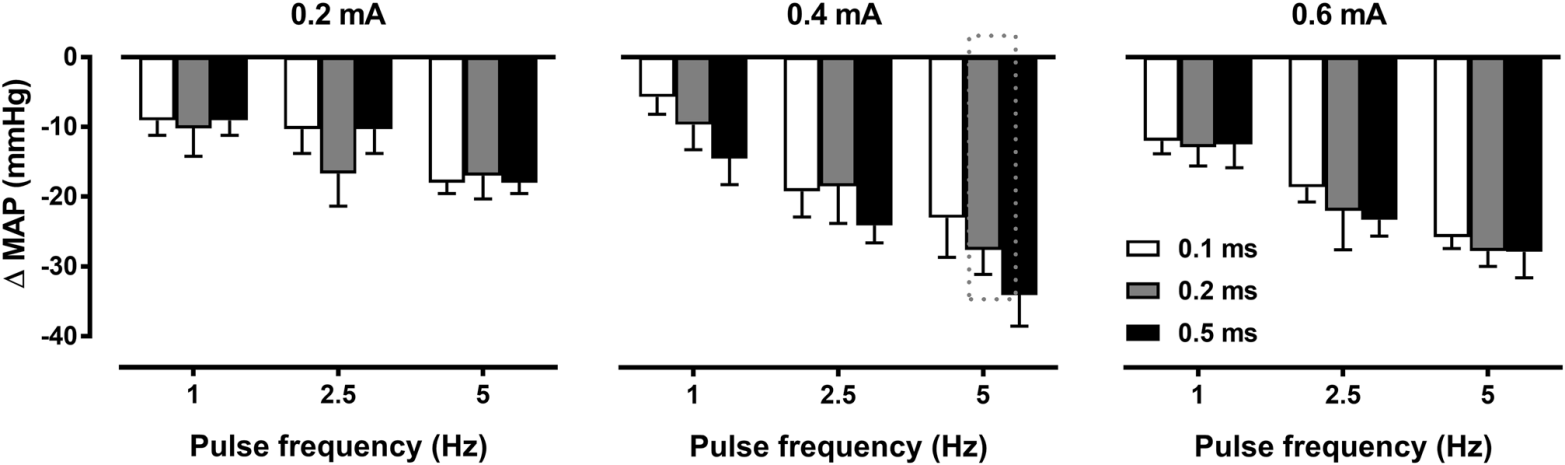
Effect of continuous (20 s) aortic depressor nerve (ADN) stimulation using low range of pulse amplitudes (0.2, 0.4 and 0.6 mA), widths (0.1, 0.2 and 0.5 ms) and frequencies (1, 2.5 and 5 Hz) on mean arterial pressure (MAP) in pentobarbital-anesthetized spontaneously hypertensive rats. Results are expressed as mean ± SEM (*n* = 4).

Baseline MAP of the SHR used in these studies was 174 ± 6 mmHg. The effect of modifying pulse width, amplitude, and frequency of ADN stimulation on MAP of SHRs is shown in Fig. 1.

A robust frequency-dependent depressor response was seen at all pulse amplitudes and widths used (*P* < 0.05 for all responses). Lower pulse amplitudes of 0.2 mA produced an average decrease in MAP of 9 ± 2 to 18 ± 2 mmHg at all pulse widths tested. Higher pulse intensity of 0.4 mA produced the largest decrease in MAP at 5 Hz stimulation frequency, with a median MAP reduction of 28 ± 4 mmHg seen at a pulse width of 0.2 ms. Higher pulse amplitude of 0.6 mA resulted in relatively similar drops in MAP to that of the of 0.4 mA at all pulse widths and frequencies tested.

### Study 2: Effect of continuous *versus* intermittent stimulation of the ADN using low (5 Hz) and high (15 Hz) stimulation frequency

Baseline hemodynamics prior to the 5 Hz and 15 Hz stimulation protocols are presented in Table 1. There was no baseline shift in the measured variables prior to each stimulation protocol. However, irrespective of the mode of stimulus delivery, both 5 Hz and 15 Hz stimulations significantly lowered MAP, HR, MBF, MVR and FVR relative to baseline. Reflex FBF, by contrast, was higher compared with baseline values.

**Table 1:** Baseline and peak hemodynamics recorded in response to continuous (CONT) and intermittent (5 s ON/3 s OFF and 5 s ON/5 s OFF) stimulation of the aortic depressor nerve (ADN) using low (5 Hz) and high (15 Hz) pulse frequencies delivered for 20 s at 0.4 mA and 0.2 ms in pentobarbital-anesthetized spontaneously hypertensive rats.

Parameter	5 Hz				15 Hz			
	Baseline	CONT	5 s ON/3 s OFF	5 s ON/5 s OFF	Baseline	CONT	5 s ON/3 s OFF	5 s ON/5 s OFF
MAP (mmHg)	171 ± 10	143 ± 11^a^	146 ± 12^a^	141 ± 12^a^	170 ± 9	117 ± 8^a^	136 ± 10^a^	134 ± 9^a^
HR (bpm)	344 ± 8	330 ± 8^a^	332 ± 7^a^	333 ± 7^a^	341 ± 7	319 ± 8^a^	329 ± 8^a^	329 ± 7^a^
MBF (ml min^−1^)	5.5 ± 0.4	4.7 ± 0.3^a^	4.8 ± 0.4^a^	4.6 ± 0.3^a^	5.7 ± 0.4	4.2 ± 0.3^a^	4.4 ± 0.3^a^	4.6 ± 0.3^a^
MVR (mmHg min ml^−1^)	32 ± 3	30 ± 3^a^	30 ± 3^a^	30 ± 3^a^	32 ± 3	26 ± 3^a^	28 ± 2^a^	28 ± 2^a^
FBF (ml min^−1^)	0.9 ± 0.1	1.1 ± 0.1^a^	1.1 ± 0.1^a^	1.1 ± 0.1^a^	0.9 ± 0.1	1.3 ± 0.1^a^	1.2 ± 0.1^a^	1.2 ± 0.1^a^
FVR (mmHg min ml^−1^)	192 ± 12	145 ± 15^a^	137 ± 11^a^	140 ± 13^a^	190 ± 14	97 ± 14^a^	138 ± 19^a^	123 ± 18^a^

The data are presented as mean ± SEM (*n* = 8–9) and were analyzed by a two-way ANOVA followed by Bonferroni’s post hoc test.

*MAP* mean arterial blood pressure, *HR* heart rate, *MBF* mesenteric blood flow, *MVR* mesenteric vascular resistance, *FBF* femoral blood flow, *FVR* femoral vascular resistance.

^a^*P* < 0.05 *versus* respective baseline levels.

**Figure 2:**
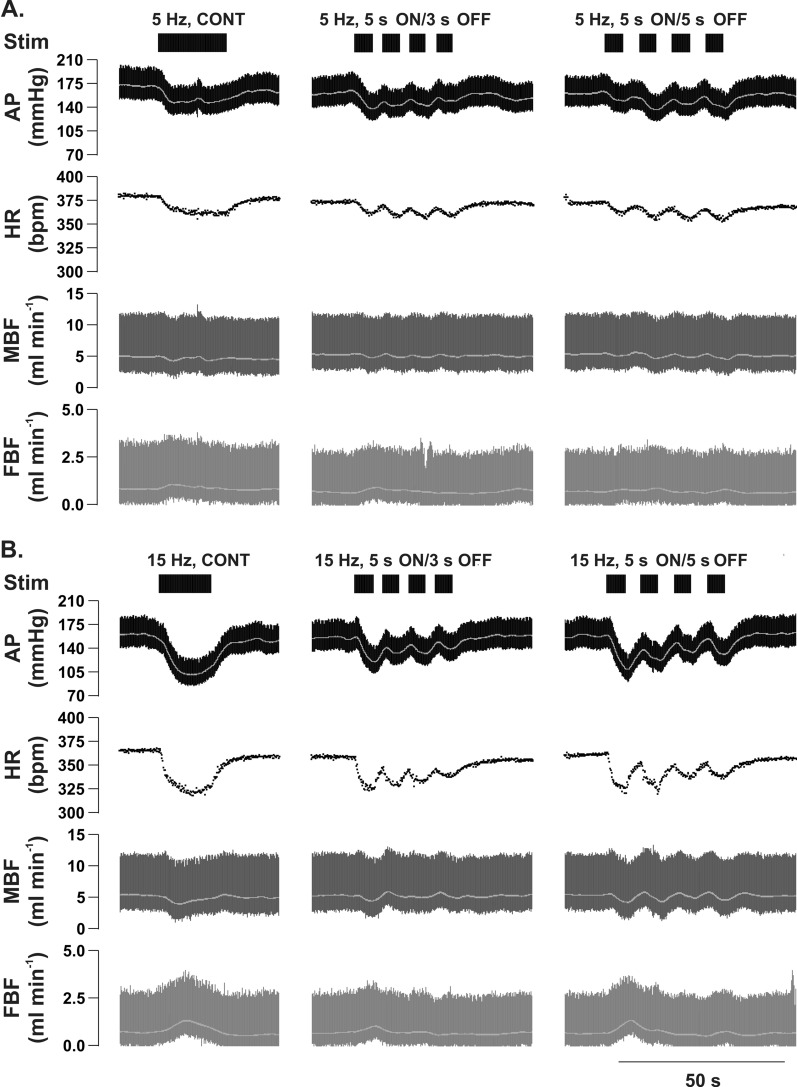
Representative raw data traces showing the effect of low (5 Hz, **A**) and high (15 Hz, **B**) frequency continuous (CONT) *versus* intermittent (5 s ON/3 s OFF and 5 s ON/5 s OFF) aortic depressor nerve (ADN) stimulation (0.4 mA, 0.2 ms for 20 s) on cardiovascular parameters in pentobarbital-anesthetized spontaneously hypertensive rats. Arterial pressure (AP), heart rate (HR), mesenteric blood flow (MBF) and femoral blood flow (FBF).

A representative raw data trace illustrating the effects of continuous and intermittent stimulation of the ADN using 5 Hz and 15 Hz stimulation frequencies in one SHR is shown in Fig. 2.

**Figure 3:**
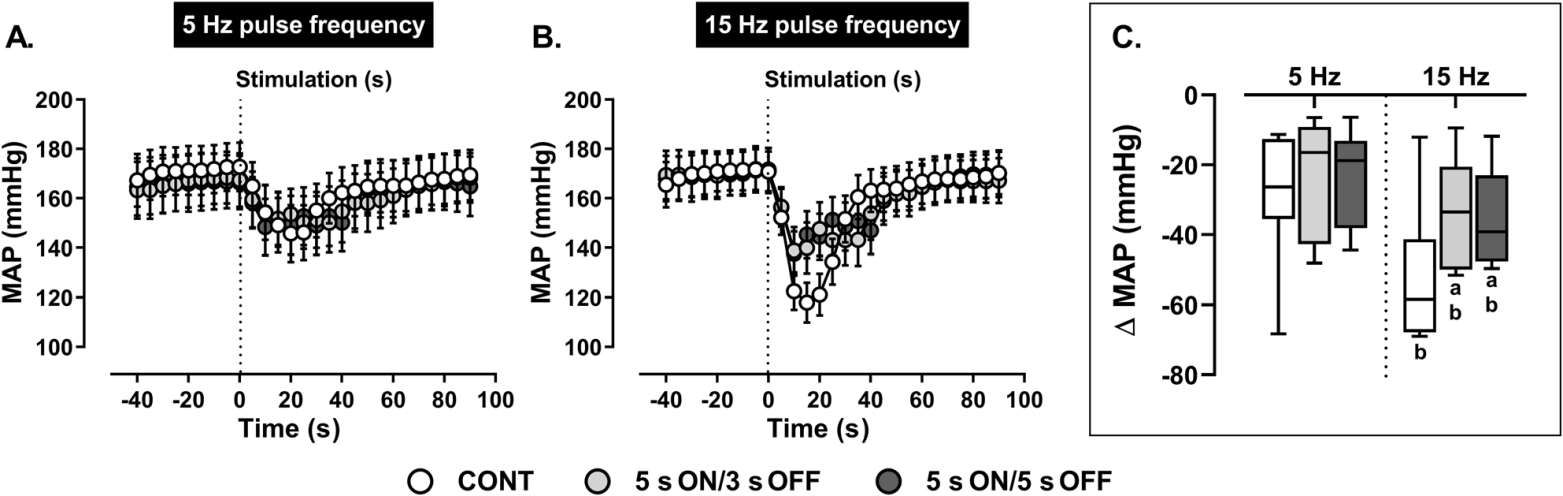
Effect of (**A**) low (5 Hz) and (**B**) high (15 Hz) frequency continuous (CONT) *versus* intermittent (5 s ON/3 s OFF and 5 s ON/5 s OFF) aortic depressor nerve (ADN) stimulation (0.4 mA, 0.2 ms for 20 s) on reflex reductions in mean arterial pressure (MAP) in pentobarbital-anesthetized spontaneously hypertensive rats. (**A,B**) The time trend for reflex depressor responses to ADN stimulation, while (**C**) shows absolute changes (mmHg) in MAP in response to ADN stimulation. Results are expressed as mean ± SEM (*n* = 9), analyzed by a two-way ANOVA followed by Bonferroni’s post hoc test. a: *P* < 0.05 *versus* continuous stimulation for each frequency and b: *P* < 0.05 *versus* respective stimulation across frequencies.

At 5 Hz, peak depressor responses to ADN stimulation (Fig. 3) and total AUC (Table 2) were similar regardless of whether the stimulation was delivered continuously or intermittently. At 15 Hz, on the other hand, lower drops in MAP (Fig. 3) and/or relatively smaller AUC values (Table 2) were observed when stimulation was intermittent as opposed to continuous. Comparing low *versus* high frequency data, continuous and intermittent stimulation at 15 Hz produced larger MAP reductions and/or AUC measures compared with respective stimulations at 5 Hz. Overall MAP recovery time from when the stimulus was applied until it was completely turned off was not influenced by the mode of stimulation or the frequency of the stimulus (5 Hz: continuous = 62 ± 6 s *versus* intermittent 5 s ON/3 s OFF = 70 ± 7 s *versus* intermittent 5 s ON/5 s OFF = 62 ± 6 s; and 15 Hz: continuous = 67 ± 8 s *versus* intermittent 5 s ON/3 s OFF = 70 ± 7 s *versus* intermittent 5 s ON/5 s OFF = 69 ± 7 s, all *P* > 0.05).

**Table 2:** Total area under the curve (AUC) for hemodynamic measures recorded in response to continuous (CONT) and intermittent (5 s ON/3 s OFF and 5 s ON/5 s OFF) stimulation of the aortic depressor nerve (ADN) using low (5 Hz) and high (15 Hz) pulse frequencies delivered for 20 s at 0.4 mA and 0.2 ms in pentobarbital-anesthetized spontaneously hypertensive rats.

Parameter (AUC)	5 Hz			15 Hz		
	CONT	5 s ON/3 s OFF	5 s ON/5 s OFF	CONT	5 s ON/3 s OFF	5 s ON/5 s OFF
MAP (mmHg s)	630 ± 148	495 ± 114	530 ± 122	1143 ± 164^b^	898 ± 163^b^	794 ± 117^a^
HR (bpm s)	258 ± 111	213 ± 57	277 ± 125	460 ± 99	376 ± 108	329 ± 104
MBF (ml min^−1^ s)	18 ± 5	14 ± 3	17 ± 4	31 ± 7	23 ± 6	19 ± 6
MVR (mmHg min ml^−1^ s)	37 ± 13	37 ± 10	34 ± 14	95 ± 21^b^	74 ± 15	94 ± 10^b^
FBF (ml min^−1^ s)	32 ± 4	32 ± 4	32 ± 4	32 ± 4	32 ± 4	32 ± 4
FVR (mmHg min ml^−1^ s)	1085 ± 408	1005 ± 316	1042 ± 326	1402 ± 262	1074 ± 331	1102 ± 263

The data are presented as mean ± SEM (*n* = 4–9) and were analyzed by a two-way ANOVA followed by Bonferroni’s post hoc test.

*MAP* mean arterial blood pressure, *HR* heart rate, *MBF* mesenteric blood flow, *MVR* mesenteric vascular resistance, *FBF* femoral blood flow, *FVR* femoral vascular resistance.

^a^*P* < 0.05 *versus* continuous stimulation for each frequency.

^b^*P* < 0.05 *versus* respective stimulation across frequencies.

**Figure 4:**
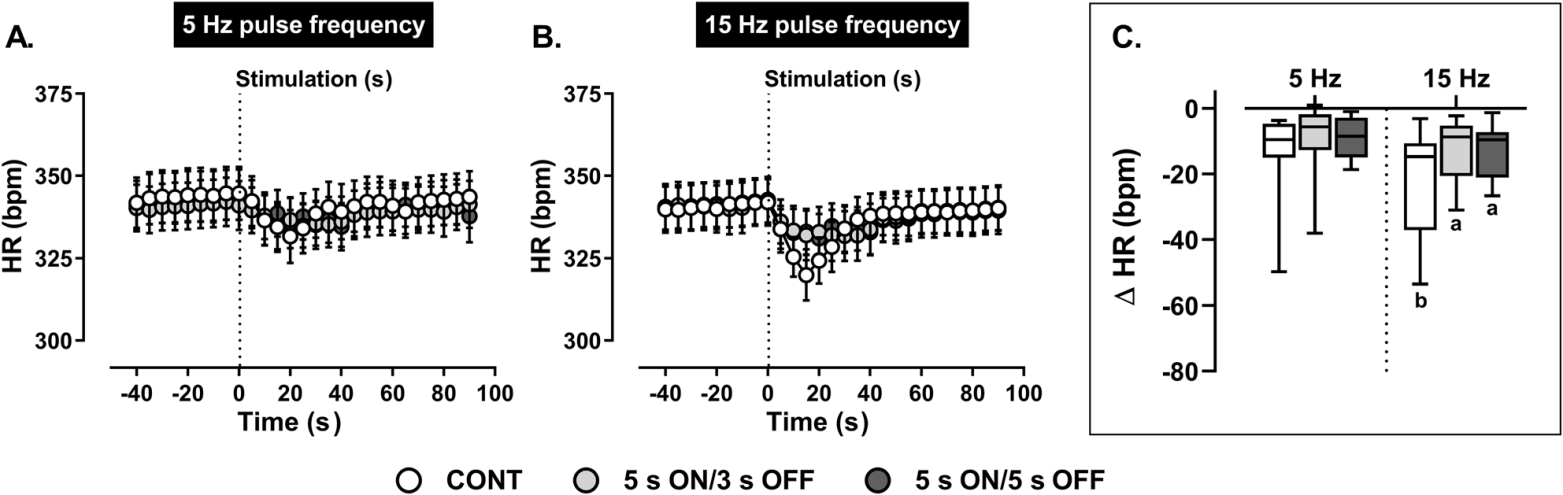
Effect of (**A**) low (5 Hz) and (**B**) high (15 Hz) frequency continuous (CONT) *versus* intermittent (5 s ON/3 s OFF and 5 s ON/5 s OFF) aortic depressor nerve (ADN) stimulation (0.4 mA, 0.2 ms for 20 s) on reflex reductions in mean heart rate (HR) in pentobarbital-anesthetized spontaneously hypertensive rats (SHRs). (**A,B**) The time trend for the reflex bradycardic responses to ADN stimulation, while (**C**) shows absolute changes (bpm) in HR in response to ADN stimulation. Results are expressed as mean ± SEM (*n* = 8), analyzed by a two-way ANOVA followed by followed by Bonferroni’s post hoc test. a: *P* < 0.05 *versus* continuous stimulation for each frequency and b: *P* < 0.05 *versus* respective stimulation across frequencies.

Irrespective of the mode of stimulus delivery, reflex bradycardic responses (Fig. 4), and their calculated AUC at 5 Hz (Table 2) did not markedly differ. High frequency (15 Hz) continuous stimulation produced the largest reflex bradycardia (Fig. 4) relative to continuous stimulation at 5 Hz and intermittent stimulations at 15 Hz. Intermittent stimulation at 5 and 15 Hz generated similar reductions in HR. Despite numerically larger AUCs for HR, values did not significantly differ within and across frequencies (Table 2).

**Figure 5:**
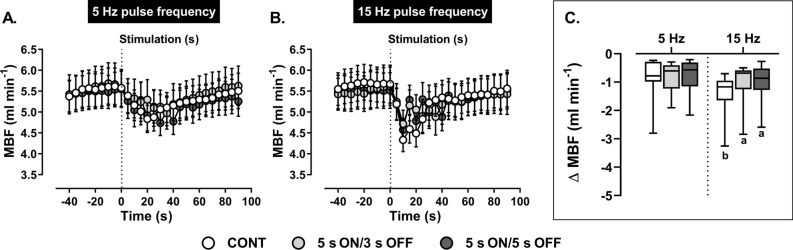
Effect of (**A**) low (5 Hz) and (**B**) high (15 Hz) frequency continuous (CONT) *versus* intermittent (5 s ON/3 s OFF and 5 s ON/5 s OFF) aortic depressor nerve (ADN) stimulation (0.4 mA, 0.2 ms for 20 s) on reflex reductions in mesenteric blood flow (MBF) in pentobarbital-anesthetized spontaneously hypertensive rats (SHRs). (**A,B**) The time trend for reflex MBF responses to ADN stimulation, while (**C**) shows absolute changes (ml min^−1^) in MBF in response to ADN stimulation. Results are expressed as mean ± SEM (*n* = 9), analyzed by a two-way ANOVA followed by followed by Bonferroni’s post hoc test. a: *P* < 0.05 *versus* continuous stimulation for each frequency and b: *P* < 0.05 *versus* respective stimulation across frequencies.

When ADN stimulation was applied continuously or intermittently at 5 Hz, reflex reduction in MBF (Fig. 5) and calculated AUC (Table 2) remained unaltered. Continuous stimulation at 15 Hz evoked the largest reflex reduction in MBF (Fig. 5) compared with continuous stimulation at 5 Hz and intermittent stimulation at 15 Hz. These findings were not associated with significant differences in AUCs. Likewise, MBF peak and AUC responses to intermittent stimulation at 5 and 15 Hz were relatively comparable.

**Figure 6:**
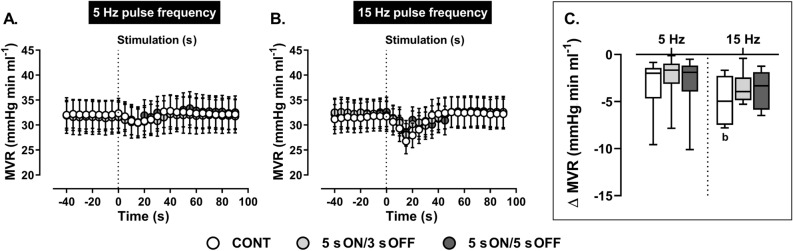
Effect of (**A**) low (5 Hz) and (**B**) high (15 Hz) frequency continuous (CONT) *versus* intermittent (5 s ON/3 s OFF and 5 s ON/5 s OFF) aortic depressor nerve (ADN) stimulation (0.4 mA, 0.2 ms for 20 s) on reflex reductions in mesenteric vascular resistance (MVR) in pentobarbital-anesthetized spontaneously hypertensive rats. (**A,B**) The time trend for the reflex MVR response to ADN stimulation, while (**C**) shows absolute changes (mmHg ml min^−1^) in MVR in response to ADN stimulation. Results are expressed as mean ± SEM (*n* = 9), analyzed by a two-way ANOVA followed by followed by Bonferroni’s post hoc test. b: *P* < 0.05 *versus* respective stimulation across frequencies.

Calculated reflex reductions in MVR (Fig. 6) in response to stimulation of the ADN at 5 Hz were similar during both continuous and intermittent stimulation. Similarly, when stimulation frequency was increased to 15 Hz peak reductions in MVR (Fig. 6) were comparable during continuous and intermittent stimulation. Across frequencies, continuous stimulation at 15 Hz caused a larger reflex reduction in MVR compared with respective stimulation at 5 Hz (Fig. 6).

Calculated reflex reductions in MVR (Fig. 6) in response to stimulation of the ADN at 5 Hz were similar during both continuous and intermittent stimulations. Similarly, when the stimulation frequency was increased to 15 Hz peak reductions in MVR (Fig. 6) were comparable during continuous and intermittent stimulation. Across frequencies, continuous stimulation at 15 Hz caused a larger reflex reduction in MVR compared with respective stimulation at 5 Hz (Fig. 6). At 15 Hz, the peak MVR responses to intermittent stimulation (Fig. 6) also tended to be greater than corresponding stimulation at 5 Hz, however these differences did not reach statistical significance (*P* = 0.07). Calculated AUCs for each frequency remained unchanged (Table 2) whereas across frequencies, 15 Hz continuous and intermittent (5 s ON/5 s OFF) stimulations generated larger AUCs for the MVR response (Table 2).

**Figure 7:**
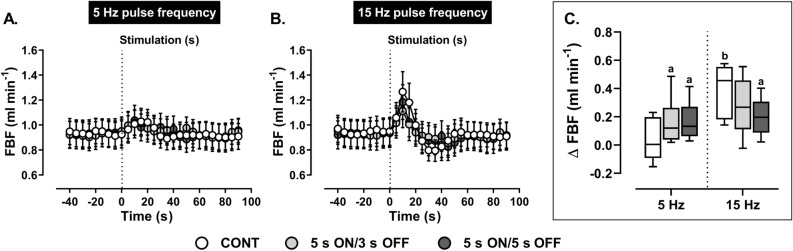
Effect of (**A**) low (5 Hz) and (**B**) high (15 Hz) frequency continuous (CONT) *versus* intermittent (5 s ON/3 s OFF and 5 s ON/5 s OFF) aortic depressor nerve (ADN) stimulation (0.4 mA, 0.2 ms for 20 s) on reflex increases in femoral blood flow (FBF) in pentobarbital-anesthetized spontaneously hypertensive rats. (**A,B**) The time trend for the reflex FBF response to ADN stimulation, while (**C**) shows absolute changes (ml min^−1^) in FBF in response to ADN stimulation. Results are expressed as mean ± SEM (*n* = 8), analyzed by a two-way ANOVA followed by followed by Bonferroni’s post hoc test a: *P* < 0.05 *versus* continuous stimulation for each frequency and b: *P* < 0.05 *versus* respective stimulation across frequencies.

Peak increases in FBF (Fig. 7), but not AUC (Table 2), in response to ADN stimulation at 5 Hz were higher using the intermittent protocol. In contrast, intermittent stimulation (5 s ON/5 s OFF but not 5 s ON/3 s OFF) of the ADN at 15 Hz (Fig. 7) caused smaller peak increases in FBF compared with continuous stimulation, with differences being most evident using 5 s ON/5 s OFF stimulation. When comparing across frequencies, continuous stimulation at 15 Hz resulted in larger FBF increases than those evoked by continuous stimulation at 5 Hz drops (Fig. 7). Intermittent stimulation, by contrast, generated relatively similar FBF responses at 5 and 15 Hz (Fig. 7). Measures of AUC did not differ across the different modes of stimulation or stimulation frequencies (Table 2).

**Figure 8:**
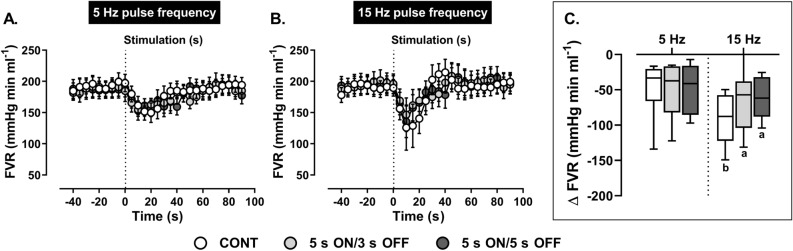
Effect of (**A**) low (5 Hz) and (**B**) high (15 Hz) frequency continuous (CONT) *versus* intermittent (5 s ON/3 s OFF and 5 s ON/5 s OFF) aortic depressor nerve (ADN) stimulation (0.4 mA, 0.2 ms for 20 s) on reflex reductions in femoral vascular resistance (FVR) in pentobarbital-anesthetized spontaneously hypertensive rats. (**A,B**) show the time trend for the reflex FVR response to ADN stimulation, while (**C**) shows absolute changes (mmHg ml min^−1^) in FVR in response to ADN stimulation. Results are expressed as mean ± SEM (*n* = 8), analyzed by a two-way ANOVA followed by followed by Bonferroni’s post hoc test. a: *P* < 0.05 *versus* continuous stimulation for each frequency and b: *P* < 0.05 *versus* respective stimulation across frequencies.

Reflex reductions in FVR at 5 Hz (Fig. 8) were similar during continuous or intermittent ADN stimulation. In contrast, maximal reductions in FVR at 15 Hz continuous stimulation (Fig. 8) were significantly greater relative to intermittent stimulation at 15 Hz and continuous stimulation at 5 Hz. Peak FVR responses to intermittent stimulation at 5 and 15 Hz (Fig. 8) as well as measured FVR AUC values across both the 5 and 15 Hz protocols (Table 2) did not markedly differ.

## Discussion

This study supports the concept that aortic baroreceptor afferents may be a viable alternative neuromodulation target to carotid baroreceptors stimulation in lowering ABP. The major finding of the present exploratory study is that in hypertension, modulation of aortic baroreceptor afferent activity in order to drive appreciable and prompt reductions in ABP does not necessarily require the use of a high charge injection system or the delivery of the stimulus in a continuous manner. This approach markedly lowers energy consumption for neuromodulation and also minimizes potential unwanted disturbances in cardiovascular variables. Clinically, these findings may therefore provide useful insight for studies using BAT in resistant hypertension and other cardiovascular diseases.

It has long been suggested that aortic and carotid baroreceptors and their afferents have different pressure sensitivities, with experimental evidence demonstrating greater mechano-transduction properties of aortic baroreceptor afferents and thus higher-pressure sensitivity than those of carotid baroreceptors^22,24^. This highlights the possibility that aortic baroreceptors may be an additional and possibly superior interventional target for ABP modulation. However, irrespective of the neuromodulation target, many, if not most, of the published clinical and experimental studies including previous studies from our laboratory assessed reflex cardiovascular responses to baroreflex activation using supramaximal stimulation parameters^27,28^. Neurophysiologists routinely expose nerves to high stimulation currents/voltages to alter recruitment patterns of A and C fibers when studying various functional aspects of nerves. While this approach defines the whole range of responses a nerve can elicit, generated outcomes are far from physiological and have limited translatability to clinical practice. Despite the effective and maintained reductions in ABP in response to BAT, the technology is still criticized for the use of ultrahigh stimulation parameters, which increases risk of field stimulation and potential off-target effects, and also impacts nerve structural integrity and promotes nerve fatigue. From a device development perspective, a supramaximal stimulus intensity does very little to inform of a device development pathway with translational applicability and an ultimate goal to treat patients for multiple disease indications. In view of that, a major consideration in this study was to use a neurostimulation model that enabled low energy consumption to characterize the cardiovascular response within it. During our first study, SHRs MAP responses were tested using different low-stimulation-range combinations of pulse intensities (0.2, 0.4 and 0.6 mA), widths (0.1, 0.2 and 0.5 ms) and frequencies (1, 2.5 and 5 Hz), with the aim of providing a clinically acceptable drop in ABP of 20 – 30 mmHg as described by others^10,16,25,26^.

When stimulated acutely in resistant hypertensive patients, device-based unilateral carotid baroreflex activation was previously shown to evoke more than 20 mmHg reduction in MAP at 4 – 7 V, 20 – 100 Hz, 0.48 ms^10^. In conscious SHRs, an approximate 30 mmHg drop in MAP was reported with electrical stimulation of the left carotid sinus nerve at 3 V, 30 Hz and 1 ms^29^. On the other hand, left ADN stimulation at 1 mA, 5 Hz and 2 ms produced an approximate 25 mmHg drop in MAP in conscious L-NAME-induced hypertensive rats, with stimulations beyond 5 Hz resulting in excessive reflex hypotensive responses^19^. Moreover, a MAP decrease of approximately 30 mmHg in conscious SHRs was attainable when the left ADN was stimulated at 1 mA, 10–15 Hz and 2 ms^18^. Remarkably, our data reproduced these ABP findings at reduced pulse intensity (0.4 mA) and lower pulse frequency (5 Hz) and/or width (0.2 ms). Our data further demonstrated “no added benefit” for currents beyond 0.4 mA in terms of driving greater reflex depressor responses to ADN stimulation. Together, these findings suggest that it is not necessary to use a high charge injection model to alter afferent traffic within the ADN. Indeed, in previous studies from our laboratory, we have frequently encountered changes in the physical outlook of nerves from bright and translucent to dull and brown because of the intense heat generated by higher pulse amplitudes, longer pulse widths and higher frequencies. From a physiological perspective, such experimental design is often questioned about the quality of data collected at high intensity stimulations, which do not reproduce the natural recruitment pattern of afferent fibers with different pressure thresholds that occur with the spontaneous increases in ABP. The high charge injection model may also represent one of many other factors that underlie the challenges faced in designing longitudinal baroreflex studies and maintaining nerve viability during chronic implant procedures as shown previously in short-term conscious studies in rats^18,19^. Clinically, selectivity, safety and efficacy issues have often been raised when charge density for BAT was set to higher threshold values. To that end, supramaximal stimulations of the carotid sinus using BAT technology has been shown to alter breath duration and breathing frequency^30^. Jaw or neck pain, swallowing sensation, coughing, or voice problems were also noted by hypertensive patients when BAT employed high stimulation intensities^9^.

In the next series of experiments, we aimed to (1) determine whether the energy required to maintain the ABP decrease in response to ADN stimulation can be minimized even further by delivering the stimulus in an intermittent rather than continuous manner; and (2) assess how altering afferent fibers recruitment pattern by modulating pulse frequency rather than pulse intensity can impact the level of the reflex depressor response. With the intermittent stimulation, a 3 – 5 s OFF period was chosen in order to avoid full recovery of ABP when the stimulus was withdrawn. As far as nerve fibers recruitment are concerned, a low frequency of 5 Hz and a higher one of 15 Hz were chosen since early work from Andresen and colleagues suggested that fibers recruitment in the ADN can be altered by using low-frequency (< 10 Hz) stimulation to engage C-fiber type or high-frequency (> 10 Hz) to trigger A-fiber type^31,32^.

During low frequency stimulation, both intermittent and continuous stimulation evoked relatively similar peak hypotensive responses of about 30 mmHg, with both modes of intermittent stimulation (5 s ON/3 s OFF and 5 s ON/5 s OFF) likewise generating comparable results. When comparing ABP recovery after both intermittent and continuous stimulation, responses were similarly sustainable throughout the stimulation window as evidenced by the comparable AUC measures and recovery time for intermittent and continuous MAP responses. Reflex bradycardic and vascular resistance responses to low frequency stimulation mirrored those of ABP, suggesting that the comparable reflex depressor responses to both intermittent and continuous stimulation were potentially driven by the similar reflex changes in HR and vascular resistance. Importantly, these results indicate that a clinically relevant drop in ABP can be evoked by solely engaging C-fiber afferents and that energy consumption during stimulation cannot only be minimized using a low charge injection system but also through intermittent delivery of the electrical stimulus.

Recruitment of A-fiber type using higher frequency stimulation induced larger immediate drops in ABP (approximately 50 mmHg), HR, MVR and FVR, however this was only apparent when the stimulus was applied continuously. Irrespective of the OFF period, intermittent high frequency stimulation, lowered the excessive reductions in ABP, HR and vascular resistances encountered with the continuous stimulation, suggesting that intermittent high frequency stimulation offers a less drastic impact on cardiovascular responses than continuous stimulation. Indeed, a major concern with large immediate reductions in ABP is the resulting hemodynamic instability that can occur with baroreflex activation^25^, which is a leading cause for impaired organ perfusion^33^.

The ABP responses to both modes of intermittent high frequency stimulation were slightly, but significantly higher and more sustained than the corresponding low frequency stimulation. This indicates that intermittent high frequency stimulation may be sought after if an additional reduction in ABP is required. Importantly, when compared to the corresponding responses to low frequency stimulation, the significant ABP responses to intermittent high frequency stimulation were not mirrored by similarly large changes in the other recorded hemodynamic variables. It is well known that changes in peripheral vascular resistance are the primary contributor to changes in ABP^34^ and while the recorded HR and vascular resistance responses would have, at least partly, prompted this ABP response, the current data suggest the involvement of some unexplored vascular beds in this study, which could have similarly contributed to the overall reduction in systemic vascular resistance in response to the stimulation.

Of note was the finding that the hemodynamic responses to 15 Hz stimulations, but less so with 5 Hz stimulations, were trending back toward baseline level particularly when the stimulus was applied intermittently. It is known that the A-type baroreceptor afferents alone are capable of separately producing 70 – 80% of the combined maximal MAP response^31^. It is therefore possible that high frequency intermittent volleys, when delivered briefly (5 s) during the ON period, were unable to achieve maximal activation of the A-type fibers, resulting in a lower blood lowering efficacy. Alternatively, the hemodynamic response pattern with high frequency may suggest acute resetting of the arterial baroreflex, which was likely driven by altered baroreceptor afferent sensitivity^35^ in response to the frequent exposure of the nerve to the electrical stimulus.

A limitation of this study was the recording of hemodynamic responses to ADN stimulation under anesthesia. Surgical anesthesia is often criticized for its unfavorable impact on cardiovascular and autonomic reflexes, and thus the use of conscious studies enables recording of cardiovascular variables with a higher fidelity. However, success of conscious longitudinal baroreflex studies using the ADN in rats is currently low and hampered by (1) inherent anatomical challenges given the small diameter of the nerve and the relative ease to sustain a damage to it during dissection and manipulation; and (2) the use of unrefined electrode design and nerve stimulation protocols that can ensure long-term viability of the nerve. To our knowledge, current studies reporting hemodynamic responses to ADN stimulation in conscious rats are typically run within 24 h after surgical implantation of the electrodes^18,29,36^. It is therefore possible that reported measures by those studies may, to a great extent, be confounded by issues related to surgical stress and inadequate animal recovery. Therefore, in order to ensure successful transition to conscious baroreflex studies, future research work should address these challenges.

A second limitation was the use of pentobarbital for induction and maintenance of anesthesia, an anesthetic known to evoke a greater suppression of hypothalamic cardiovascular regulation and baroreflex reinforcement^37^ relative to other forms of anesthesia including urethane and inhalation anesthetics. While urethane^38^ and inhalation anesthetics (e.g., isoflurane, sevoflurane or enflurane)^39^ have less impact on baroreflex control, owing to their considerable reduction of sympathetic contribution to ABP maintenance^40^, they seem to overly lower baseline blood pressure levels in the SHRs, with values almost approaching the normotensive range^40,41^. In our hands (despite titrating urethane or isoflurane to the lowest effective dose), our SHRs, when maintained on these anesthetics, had MAP levels ranging between 80 and 100 mmHg (data not shown). These readings do not reflect the hypertensive state of the SHRs and also defeat the purpose of the model and the research question from this study. It is well known that recruitment pattern of individual baroreceptor fibers and baroreflex resetting, which can occur almost instantaneously^35^, are tied to baseline blood pressure levels and can alter the magnitude of responses to baroreflex activation. We therefore believe that low MAP baseline and resultant changes in baroreflex function were major disqualifiers for urethane and isoflurane as choices of anesthesia in the SHRs. With pentobarbital, on the other hand, baseline MAP levels were not only preserved but also matched those reported in conscious SHRs^40,42^. Importantly, pentobarbital anesthesia appears to influence baroreflex control of heart rate mainly through impairing reflex cardiac parasympathetic activity^39,42^. This also leaves reflex sympathetic control of vascular resistance, the primary determinant of blood pressure control^34^, unaffected, suggesting a relative reliability of hemodynamic data generated under pentobarbital anesthesia and other barbiturate anesthetics as shown previously^24,43,44^.

A third limitation of the current study is that hemodynamic responses to ADN stimulation were tracked briefly and thus further studies, perhaps in a reliable chronic setup, are required to assess MAP adaptation to the applied stimulus. However, based on our current findings in hypertension and those reported by others in normotensive rats^45^, we believe that sustaining a reduction in blood pressure following the withdrawal of the electrical stimulus is unlikely. Whether longer stimulations can result in a baseline shift in MAP remains unclear. Preliminary evidence at least in conscious normotensive rats argues against this possibility^45^, mirroring our data by demonstrating a similar rebound in the blood pressure response toward baseline despite using a higher frequency (30 Hz) and longer stimulation protocol (60 min). However, whether different findings can be identified under hypertensive conditions remains to be determined. In the light of the above evidence, we believe that targeting the recruitment pattern of A-type *versus* C-type fibers during stimulation carries a huge potential to identify optimal stimulation parameters that can evoke a maintained drop in blood pressure. Indeed, the low frequency protocol described here offer a more consistent drop in blood pressure, with a minimal rebound seen irrespective of the continuous or intermittent mode of stimulus delivery. This indicates that low-frequency protocols (< 10 Hz) targeting the C-fiber afferents may potentially offer a more robust and sustainable drop in blood pressure in the long term as opposed to high-frequency stimulation protocols that predominantly recruit A-fiber afferents.

Finally, it can be argued that the use of rectangular impulses in our experiments may have affected nerve viability and thus skewed the data generated from the stimulation protocol. We, however, strongly believe that such possibility is quite unlikely due to 3 main reasons: (1) the short nature of our stimulations significantly lowered nerve exposure to the rectangular pulses; (2) the stimulation protocol itself implemented low charge injections with ultralow stimulation parameters; and (3) during physical inspection of the nerve throughout the experiments no gross changes in the physical outlook of the nerve was seen as the nerve maintained its bright and translucent appearance.

## Perspectives

Research on carotid baroreflex activation and the fundamental implementation of this technique as a treatment modality for hypertension has come a long way since 1958^46^. Over the past 20 years, substantial attention has been given to the *Rheo Hypertension Therapy System* and most recently, the *Barostim Neo System*, which proved effective in chronically suppressing sympathetic activity and lowering ABP. While this approach is strongly supported by experimental and clinical studies, critical endpoints for efficacy and safety have yet to be achieved, necessitating modifications in device technology and the design of better testing protocols. Current clinical research data pertaining to “carotid” BAT still report some variability in the ABP response across patients and the procedure is often criticized for concomitant chemoreflex activation especially when approaching higher stimulations^9,30^. This off-target action is believed to (1) reduce the ABP lowering efficacy of BAT in some patients given the sympathetic stimulation associated with chemoreflex activation^9^, and (2) underlie reported breathing adverse effects associated with the procedure^30^. This calls for reassessment of current BAT stimulation protocols to fine-tune them, or alternatively seeking other potential ABP modulation targets and analyzing their response pattern. Our study emphasizes the significance of “aortic” baroreceptor afferent nerves as a prospective target and extends beyond that to show how low intensity stimulation is an effective alternate way to modulate afferent traffic within baroreceptor neurons that enables low energy consumption for neuromodulation. We believe that these results will open new exploratory avenues for translational research within this field and will help guide future practices relating to the selection of stimulation parameters for the baroreceptor afferents studies in human and experimental animals.

## Materials and methods

### Animals

All surgical procedures and experimental protocols strictly followed National Institute of Health (NIH) guidelines, were approved by the Institutional Animal Care and Use Committee of Case Western Reserve University and were in accordance with the recommendations in the ARRIVE guidelines (https://arriveguidelines.org/). Adult male SHRs (*n* = 13, body weight = 320–390 g) were sourced from Envigo (Indianapolis, IN, USA). Four SHRs were used in Study 1, which aimed to optimize the neurostimulation parameters and the magnitude of ABP drop. A second cohort of 9 SHRs was used in Study 2, which aimed to assess cardiovascular responses to continuous and intermittent nerve stimulation. Rats were kept under controlled temperatures (22 °C) and constant 12:12 h light–dark cycle and received food and water ad libitum.

### Surgical procedure

All rats were anesthetized with an intraperitoneal injection of 50 mg kg^−1^ of sodium pentobarbital (Diamondback, Arizona, USA) and the depth of anesthesia was evaluated by testing withdrawal reflexes to toe pinch before initiating any surgery. Core body temperature was maintained using a thermostatically controlled heating blanket (T/Pump warm water re-circulator, Stryker Medical, Michigan, USA). The right femoral artery was catheterized to record ABP, and the right femoral vein was cannulated to deliver a continuous infusion (Harvard Apparatus Ltd., Massachusetts, USA) of the maintenance anesthetic (10 mg kg^−1^ h^−1^ of pentobarbital sodium in saline administered at 2 ml h^−1^). A ventral neck incision was made to expose the trachea and the left ADN. A tracheotomy was performed to facilitate spontaneous breathing. A 4–6 mm segment of the ADN was isolated distal to the point where it entered the superior laryngeal nerve and placed on bipolar silver-wire stimulating electrodes (inter-electrode distance of approximately 1 mm) and maintained uncut in mineral oil, as described previously^27,28,47^. The stimulating electrodes were then connected to a square pulse stimulator (S88 Dual Output Square Pulse Stimulator, Grass Technologies Product Group, Wisconsin, USA) using a stimulus isolation unit (Grass Instrument Co. Model PSIU6 Photoelectric Stimulus Isolation Unit, Grass Technologies Product Group, USA) to deliver monophasic electrical current. All corresponding voltage traces were recorded using a digital oscilloscope (Yokogawa Digital Oscilloscope DL708E, Tokyo, Japan).

In some rats (Study 2), the superior mesenteric artery and the left femoral artery were dissected free and a clip-on-type flow probe (TS420 Perivascular Flow Module, Transonic Systems Inc., New York, USA) was placed around them for continuous concomitant recording of mesenteric and femoral blood flow from both vascular beds. All recordings were acquired using CED 1401 data acquisition system (Power3A CED 1401, Cambridge Electronic Designs Ltd., Cambridge, UK) and displayed on the computer using Spike2 software (v8, Cambridge Electronic Designs Ltd.). At the end of the surgical procedure, animals were allowed to stabilize for 15–30 min before commencing experimental protocols.

## Experimental protocol

### Study 1

Peak MAP responses elicited by 20 s stimulations of the left ADN delivered continuously using low pulse amplitudes (0.2, 0.4 and 0.6 mA), widths (0.1, 0.2 and 0.5 ms) and frequencies (1, 2.5 and 5 Hz) were recorded. MAP was allowed to return to baseline pre-stimulus levels for 2–3 min before application of the next stimulus.

### Study 2

The changes in MAP, heart rate (HR), mesenteric blood flow (MBF) and femoral blood flow (FBF) in response to continuous (20 s) and intermittent (5 s ON/3 s OFF and 5 s ON/5 s OFF for 20 s) stimulation of the ADN were recorded at low (5 Hz) and high (15 Hz) pulse frequencies and with all stimulations delivered at 0.4 mA and 0.2 ms. All variables were allowed to return to baseline pre-stimulus levels for 2 – 3 min before applying the next stimulus.

### Data analysis

MAP and HR were derived from the arterial pressure waveform. Calculation of mesenteric (MVR) and femoral (FVR) vascular resistance were performed using the following equation^47,48^.

$${\varvec {Vascular \, Resistance}}\left( {{\text{mmHg\, min\, ml}}^{{ - {1}}} } \right) \, = {\varvec {MAP}} \left( {{\text{mmHg}}} \right)/ {\varvec {Blood \, Flow}} \left( {{\text{ml \, min}}^{{ - {1}}} } \right)$$

Baseline MAP, HR, MBF, FBF, MVR and FVR were measured over a 40 s period immediately prior to commencing the stimulation protocol. All reflex hemodynamic responses to ADN stimulation were converted into 5 s bins and plotted against time as 40 s baseline and 80 s from when the stimulus was applied. Absolute changes in MAP, HR, MBF, FBF, MVR and FVR were then calculated by measuring the peak changes in response relative to the immediate 40 s baseline prior to the application of each electrical stimulus. MAP recovery time was calculated as the time between the start of the stimulation and the time when the response was back to no more than 5% of baseline levels. Areas under the curve (AUC), reflective of the sustainability of the responses, were also calculated over a 40 s period from the start of stimulation.

### Statistics

All data are expressed as mean ± standard error of the mean (SEM). Statistical analyses were performed using GraphPad Prism software (v8, GraphPad Prism Inc., La Jolla, California, USA). A two-way ANOVA followed by Bonferroni correction for multiple comparisons between means was used to identify differences in basal values, peak responses, and AUC measures. Significance was defined as *P* ≤ 0.05.

## Data Availability

Due to confidentiality agreements, the datasets generated during and/or analyzed during the current study are not publicly available but are available from the corresponding author on reasonable request.
